# SARS-CoV-2 Variants, Vaccines, and Host Immunity

**DOI:** 10.3389/fimmu.2021.809244

**Published:** 2022-01-03

**Authors:** Priyal Mistry, Fatima Barmania, Juanita Mellet, Kimberly Peta, Adéle Strydom, Ignatius M. Viljoen, William James, Siamon Gordon, Michael S. Pepper

**Affiliations:** ^1^ Department of Immunology, Institute for Cellular and Molecular Medicine, University of Pretoria, Pretoria, South Africa; ^2^ South African Medical Research Council (SAMRC) Extramural Unit for Stem Cell Research and Therapy, Faculty of Health Sciences, University of Pretoria, Pretoria, South Africa; ^3^ James and Lillian Martin Centre, Sir William Dunn School of Pathology, University of Oxford, Oxford, United Kingdom; ^4^ Graduate Institute of Biomedical Sciences, College of Medicine, Chang Gung University, Taoyuan City, Taiwan; ^5^ Sir William Dunn School of Pathology, University of Oxford, Oxford, United Kingdom

**Keywords:** coronavirus, immunity, SARS-CoV-2, spike protein, vaccines, variants of concern

## Abstract

Severe acute respiratory syndrome coronavirus 2 (SARS-CoV-2) is a new beta coronavirus that emerged at the end of 2019 in the Hubei province of China. SARS-CoV-2 causes coronavirus disease 2019 (COVID-19) and was declared a pandemic by the World Health Organization (WHO) on 11 March 2020. Herd or community immunity has been proposed as a strategy to protect the vulnerable, and can be established through immunity from past infection or vaccination. Whether SARS-CoV-2 infection results in the development of a reservoir of resilient memory cells is under investigation. Vaccines have been developed at an unprecedented rate and 7 408 870 760 vaccine doses have been administered worldwide. Recently emerged SARS-CoV-2 variants are more transmissible with a reduced sensitivity to immune mechanisms. This is due to the presence of amino acid substitutions in the spike protein, which confer a selective advantage. The emergence of variants therefore poses a risk for vaccine effectiveness and long-term immunity, and it is crucial therefore to determine the effectiveness of vaccines against currently circulating variants. Here we review both SARS-CoV-2-induced host immune activation and vaccine-induced immune responses, highlighting the responses of immune memory cells that are key indicators of host immunity. We further discuss how variants emerge and the currently circulating variants of concern (VOC), with particular focus on implications for vaccine effectiveness. Finally, we describe new antibody treatments and future vaccine approaches that will be important as we navigate through the COVID-19 pandemic.

## Introduction

Coronaviruses (CoVs) are a diverse family of enveloped single-stranded RNA viruses that infect various vertebrates. There are four genera of CoVs (alpha, beta, gamma, and delta), with alpha and beta having the ability to cause disease in humans by crossing animal-human barriers and becoming human pathogens (1). Three highly pathogenic strains of beta CoVs with high mortality have emerged during the past two decades as a result of zoonotic transmission. The first two, severe acute respiratory syndrome virus (SARS-CoV-1) and the Middle Eastern respiratory syndrome virus (MERS-CoV) emerged in 2002 and 2012 respectively (1). SARS-CoV-2 is a new beta CoV that emerged at the end of 2019 in the Hubei province of China and causes coronavirus disease 2019 (COVID-19) (1, 2). The widespread transmission of the virus to all countries across the globe resulted in COVID-19 being declared a pandemic by the World Health Organization (WHO) on 11 March 2020 (3). As of 22 November 2021, there have been a total of 256 966 237 reported SARS-CoV-2 infections and 5 151 643 related deaths in over 100 countries around the world (4). The devastating impact of COVID-19 over the past year has resulted in a global effort to establish herd or community immunity, which begins with immunity at the individual level that will eventually scale up to population level (5).

Viral immunity is mediated by immunological memory that develops after a primary immune response has been elicited to a viral antigen. Natural SARS-CoV-2 immunity can develop from prior infection; this will result in a rapid and effective immune response, thereby protecting the host. However, the presence and duration of SARS-CoV-2-specific immune memory cells conferring reliable protective immunity in individuals with past infection remains poorly understood. Information regarding these responses could assist in determining whether naturally acquired immunity will effectively contribute to the development of herd immunity.

Active immunization can also generate herd immunity and various newly formulated, US Food and Drug Administration (FDA) approved COVID-19 vaccines are being rolled-out. The majority of the COVID-19 vaccines, such as the messenger RNA (mRNA; Pfizer-BioNTech Comirnaty - BNT162b2 & Moderna - mRNA-1273), protein-based (Novavax – NVX-CoV2373) and viral vector-based (Johnson & Johnson Janssen - Ad26.COV2.S, Oxford-AstraZeneca - AZD1222/ChAdOx1, Sputnik V - Gam-COVID-Vac-rAd26/rAd5) vaccines, primarily target the spike (S) protein, while traditional inactivated vaccines (Sinopharm - BBIBP-CorV, Sinovac - CoronaVac, Covaxin – BBV152) target the entire virus (6). As of 21 November 2021, 7 408 870 760 vaccine doses have been administered worldwide (4), but much of Africa has still not been vaccinated as the bulk of vaccine dissemination has largely been in developed nations (7).

The emergence of SARS-CoV-2 variants further limits the success of vaccines and natural immunity as they contain genomic alterations, particularly in the S protein coding regions, that increase their fitness in comparison to previously circulating strains. The SARS-CoV-2 S protein is one of four major viral structural proteins and consists of two subunits, namely S1 and S2. The N-terminal S1 subunit contains the species-specific receptor binding domain (RBD) and the majority of the fitness enhancing amino acid changes observed in circulating variants are found within this domain. The SARS-CoV-2 S protein structure is discussed in more detail in Section 2 of this article. While countries such as South Africa have suspended the administration of vaccines that fail to provide reliable protection against circulating variants, infections continue to rise across the globe. Therefore, understanding how these variants emerge as well as their impact on existing S protein-targeted vaccine therapies could lead to updated improved vaccine formulations and treatment regimens aimed at curbing the spread of the virus.

Our goal is to review the SARS-CoV-2 natural and vaccine-induced immune responses, the effect of currently circulating variants on the efficacy of COVID-19 vaccines and immunity, as well as future therapeutic strategies.

## 1 Natural vs. Vaccine-Induced Immunity to SARS-CoV-2

### 1.1 Viral Immune Response

#### 1.1.1 Innate Immune Response

**Figure 1:**
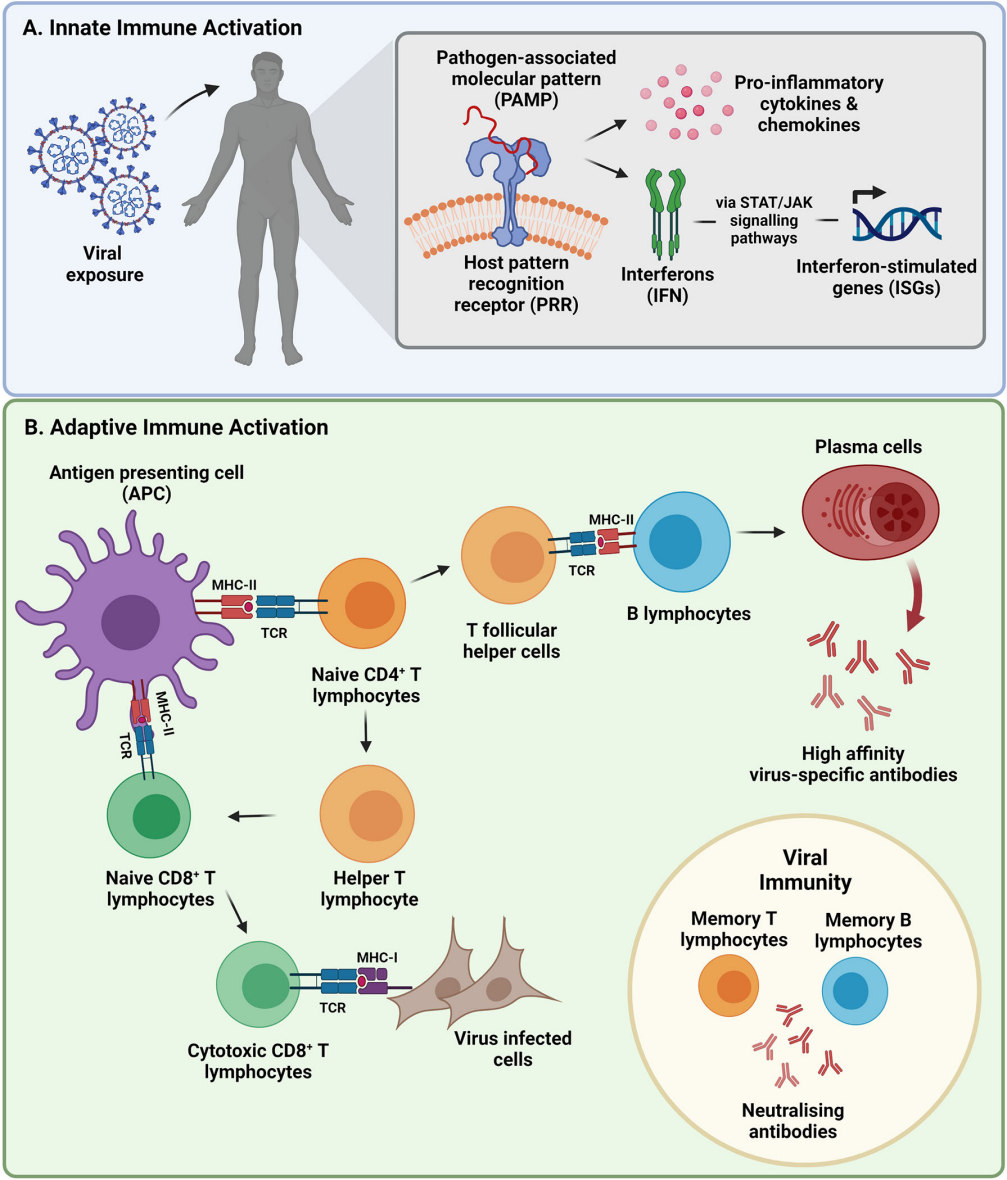
Summary of the two branches of the immune system activated during viral infection, followed by the development of viral immunity. **(A)** The innate immune response is activated within hours of viral exposure as the body’s first line of defense that releases a series of anti-viral molecules at the site of infection. **(B)** An adaptive immune response is initiated after being primed by components of the innate immune system to initiate pathogen-specific cellular and humoral immune responses days after infection. Primed immune memory cells remain following viral clearance, constituting the presence of immunity. (STAT, signal transducer and activator of transcription; JAK, Janus kinase; CD, cluster of differentiation; MHC, major histocompatibility complex; TCR, T-cell receptor). Image created by PM using BioRender (https://biorender.com/).

The innate immune response is the body’s first line of defense that plays a pivotal role in viral detection and control. SARS-CoV-2 initially infects the upper respiratory tract where early non-specific physiochemical factors such as mucus barriers trap and eliminate virus (8, 9). Mucus barriers are secreted by mucosal epithelial cells that form the inner lining of the respiratory tract and contain many pathogen defense compounds such as mucins, defensins, histatins and protegrins (10). If this protective layer is breached, innate immune sensors called pattern recognition receptors (PRRs) recognize pathogen-associated molecular patterns (PAMPs) thereby initiating the release of innate immune proteins within hours of viral exposure (11, 12) (**Figure 1**). Like other coronaviruses, SARS-CoV-2 RNA is detected by endosomal Toll-like receptor (TLR)-2, TLR3 and TLR7 (12) or cytosolic retinoic acid-induced gene 1 (RIG-1) and melanoma differentiation-associated gene 5 (MDA5) (13). It has been reported that SARS-CoV-2 infection can also be detected through the cytosolic DNA sensing cyclic GMP-AMP synthase (cGAS)-stimulator of interferon genes (STING) pathway (14). Viral detection triggers the activation of various transcription factors resulting in the secretion of pro-inflammatory chemokines and cytokines such as tumor necrosis factor alpha (TNF-α), interleukin (IL)-1 and IL-6, amongst others, by monocytes, macrophages, neutrophils, and dendritic cells (DCs) that home to the site of infection (12, 13, 15). The release of these cytokines also stimulates natural killer (NK) cells that are responsible for directly killing virus-infected cells through degranulation and receptor mediated apoptosis (16). Antiviral responses are amplified by inducing the expression of type I interferons (IFN) and subsequent interferon-stimulated genes (ISGs) (12, 13, 15).

Early release along with adequate production and proper localization of effector cells and antiviral cytokines is associated with successful control of SARS-CoV-2 (17). However, dampened type I IFN responses and elevated levels of antiviral cytokines resulting in the cytokine release syndrome (CRS) or ‘cytokine storm’ have been reported in patients with severe COVID-19 (13, 18, 19). Emphasis has therefore been placed on investigating how aberrant innate immune responses may drive the immunopathology of COVID-19. Ineffectual type I interferon responses have been attributed to immune manipulation mechanisms employed by SARS-CoV-2 to suppress host antiviral responses and enhance viral entry (17, 18, 20). Monocytes, macrophages, neutrophils, NK cells, and DCs have been implicated as key contributors to the hyperinflammation observed in COVID-19 patients with severe disease. The dysregulated function of these cells that results in over-production of cytokines and the viral mechanisms leading to these outcomes has been extensively reviewed (18, 21–26). However, their contribution to the acute innate response that leads to successful SARS-CoV-2 control remains poorly understood.

#### 1.1.2 Adaptive Immune Response

Despite being activated a few days after viral exposure, the adaptive immune response is the second arm of the host immune system that is crucial for virus elimination. Components of the innate immune system are required to stimulate and prime core cellular and humoral effector cells. Type I IFN responses induce the maturation of DCs, monocytes and macrophages into antigen presenting cells (APCs) that display viral antigenic peptides complexed with major histocompatibility complex (MHC) class II (27–30). NK cells conduct active cross-talk with DCs and play a role in antigen-presentation (16). APCs facilitate the activation of naïve cluster of differentiation (CD)4^+^ and CD8^+^ T-lymphocytes as well as regulatory T-lymphocytes (Treg) through T-cell receptor (TCR) binding (27–30) (**Figure 1**). A pattern of antigen immunodominance in convalescent COVID-19 donors has been identified with nine viral proteins being responsible for 83% of the total CD4^+^ T-lymphocyte response, while eight viral proteins account for 81% of the total CD8^+^ T-lymphocyte response (31). Some of these viral proteins included the S, nucleocapsid (N) and membrane (M) proteins as well as multiple non-structural proteins (31). In patients with severe COVID-19 disease, the total number of APCs and NK cells is however reduced resulting in suppressed antigen presenting potential (32, 33). T-cell lymphopenia, particularly the CD4^+^ T-lymphocyte subset, and an increased neutrophil to lymphocyte ratio (NLR) (indicator of inflammation) has also been observed in COVID-19 patients and correlates with disease severity (34). This may be a consequence of virus-induced suppression of the type I IFN response (35) and an uninhibited cytokine response leading to inflammatory activation of innate effector cells that negatively impact subsequent T-lymphocyte activation (36). Disruption of the synergy between the innate and adaptive immune system may therefore lead to a poorer outcome.

The humoral immune response to SARS-CoV-2 infection is mediated by antibodies elicited against epitopes present on the S and N proteins (37, 38). Antibodies directed against the RBD of the S protein demonstrate neutralizing ability and SARS-CoV-2 neutralizing immunoglobulins (Ig)A, IgM and IgG have been identified in COVID-19 patients (39) with IgM being produced first (38, 40). IgA antibodies are predominantly secreted by mucosa-associated lymphoid tissue (MALT) found in the respiratory tract where they prevent the binding of SARS-CoV-2 to the mucosal epithelium (41). The presence of IgA is crucial in SARS-CoV-2 infection as a deficiency of anti-SARS-CoV-2 IgA and secretory IgA could exacerbate severe COVID-19 infection or result in delayed viral shedding (42). These SARS-CoV-2-specific neutralizing antibodies however display a low level of somatic hypermutation (43). Naïve B-lymphocytes, under the influence of T follicular helper (Tfh) cells, proliferate and undergo somatic hypermutation to increase their antibody affinity within lymphoid microenvironments known as germinal centres (GCs) (44, 45). It is speculated that a deficiency in somatic hypermutation may be attributed to the sub-optimal differentiation of Tfh cells due to changes in the cytokine milieu, and that this results in the absence of germinal centers and a marked reduction in germinal B-lymphocytes as is observed in lymph nodes and the spleen during acute COVID-19 infection (46). Whether these alterations have an impact on the development and resilience of long-term SARS-CoV-2 humoral immunity remains to be determined.

Apart from binding viral antigens, antibodies also interact with NK cells *via* CD16 Fc-receptor (FcR) binding, and this triggers an antibody-dependent cell-mediated cytotoxicity (ADCC) response (16). Cytotoxic cellular responses are further driven by CD8^+^ T-lymphocytes that identify and directly kill infected cells by releasing soluble cytotoxic factors (perforin and granzymes) (30, 47). Although CD8^+^ T-lymphocytes are abundant in the lung tissue of COVID-19 patients with mild symptoms, increased production of perforin and granzyme B is only observed in patients with severe disease along with the expression of exhaustion markers such as PD-1 (28, 30). Activation of these markers could indicate hyperactivation or functional exhaustion (48), but the exact implication is yet to be understood. A strong CD4^+^ T-lymphocyte response has also been associated with effective control and eradication of SARS-CoV-2 through activation of other adaptive immune cells (49, 50), however, these cells show a milder activation compared to CD8^+^ T-lymphocytes (51).

Both innate and adaptive immune systems are therefore activated during SARS-CoV-2 infection, and these responses influence the pathogenesis of COVID-19. The current understanding of immunity to SARS-CoV-2 is predominantly based on blood serum and convalescent plasma analysis as discussed in this review, but several aspects such as how B-and T-lymphocytes respond to the varying stages of disease along with viral load remain unclear. Furthermore, taking a closer look at the tissue immune response to SARS-CoV-2 may provide more insight into virus-host interactions that could facilitate the development of COVID-19 therapeutic options.

### 1.2 SARS-CoV-2-Induced Memory Cells

Once infection clears, the majority of activated immune effector cells die off and a small proportion of longer-lasting memory cells remain constituting immunity (6). These are generally B- and T-lymphocyte memory cells that are highly reactive to antigenic proteins and that are pre-programmed to generate virus-specific neutralizing antibodies and effector cells, ultimately eliciting a more robust immune response should reinfection occur. Dan et al (52) identified both B- and T-lymphocyte memory cells in most individuals between five to eight months post COVID-19 infection. The magnitude of the immune memory generated from natural SARS-CoV-2 infection may however be associated with disease severity. Both memory CD4^+^ and CD8^+^ T-lymphocyte frequencies were higher in non-hospitalized COVID-19 patients whereas memory B-lymphocyte frequencies were higher in hospitalized patients (52). Interestingly, pre-existing memory CD4^+^ and CD8^+^ T-lymphocytes potentially effective against SARS-CoV-2 were found in people with no history of COVID-19 infection or vaccination (53). These reactive T-lymphocytes may have originated from previous exposure to other Beta CoVs that causes the ‘common cold’ (54). Although more in-depth studies are needed to further investigate the role of pre-existing memory T-lymphocytes against SARS-CoV-2, immune cross-reactivity from other previously circulating CoVs could potentially boost host immunity to SARS-CoV-2.

Patients who had recovered from mild COVID-19 infection were found to have developed quiescent long-lived bone marrow plasma cells (55). These plasma cells were able to persistently give rise to S protein-specific antibodies (55). Hartley et al *(* 56) found that patients developed B-lymphocyte memory cells against either the S or N proteins of SARS-CoV-2 and that this was stable eight months after infection. Similarly, it was found that B-lymphocyte memory cells were stable for six months after infection and accumulated more in patients with severe disease with IgG being the most dominant isotype (52, 57, 58). However, Gaebler et al. (59) found that B-lymphocyte memory cells do not decay after six months but rather evolves and can mount an effective response against the virus upon re-exposure (59). Therefore, although neutralizing antibodies decay over time (59), the continuous maturation of B-lymphocyte memory cells and plasma cells may result in production of neutralizing antibodies upon reinfection.

### 1.3 SARS-CoV-2-Induced Neutralizing Antibodies

Circulating neutralizing SARS-CoV-2 antibodies are a major contributor to protective immunity (60). Approximately 90% of SARS-CoV-2 positive immunocompetent individuals develop anti-SARS-CoV-2 antibodies (61), and COVID-19 patients with asymptomatic or mild disease have been observed to have lower neutralizing antibody titers than patients with severe disease (62–64). Predictive models have estimated that 19.9% of the mean neutralizing antibody titer found in convalescent serum is required to achieve a 50% protective neutralization level (60). It remains unclear however whether these low antibody levels reach the threshold required to confer long-term immune protection (64). Various reports have also indicated that some individuals who contracted COVID-19 did not generate antibodies at all, suggesting that the innate immune response is sufficient to eliminate the virus (65, 66). These patients may therefore not possess the necessary immune footprint required for SARS-CoV-2 immunity, implying that protective immunity from natural SARS-CoV-2 infection may not always be guaranteed.

### 1.4 Vaccine-Induced Immune Responses to SARS-CoV-2

COVID-19 vaccine mediated immune activation is intended to mimic that of natural SARS-CoV-2 infection in order to develop the same effector and memory subsets but without infecting the host or triggering severe inflammatory side effects (67). Teijaro et al (67) provide an overview as to how the various COVID-19 vaccines elicit an immune response and consequent immunity to SARS-CoV-2. Vaccines generally contain an immunogen that encodes antigenic viral peptides and an adjuvant that triggers a highly orchestrated immune response (68, 69). Depending on the vaccine formulation, the immunogen can function as both the immunogen and the adjuvant (67).

During clinical trials, COVID-19 mRNA vaccines were found to induce the maturation of CD4^+^ and CD8^+^ T-lymphocytes (70) and more than 70% of vaccinated individuals have memory T-lymphocyte responses (71). Similarly, individuals who received COVID-19 mRNA vaccines developed B-lymphocytes and high levels of IgM and IgG antibodies which were detected eight weeks after the second dose (72). Moreover, RBD memory B-lymphocyte levels were equivalent to those found in individuals who had acquired antibodies from natural SARS-CoV-2 infection (72). Recent evidence suggests that COVID-19 mRNA vaccine-induced memory T-lymphocyte and B-lymphocyte levels remain relatively stable for 3-6 months post-vaccination (73). Consistent with Phase II results (74), the Oxford-AstraZeneca (AZD1222/ChAdOx1) viral-vector based vaccine induced S protein-specific T-lymphocyte responses that peaked at 14 days after the first vaccine dose during Phase III trials (75). The Sputnik V (Gam-COVID-Vac-rAd26/rAd5) viral-vector based vaccine, that employs a two-dose prime-boost regimen, also induced robust S protein-specific cellular and humoral responses during Phase III trials (76). The durability and magnitude of S protein-specific B- and T-lymphocyte memory cells remains poorly understood as population studies are lacking.

### 1.5 Vaccine-Induced Neutralizing Antibody Responses to SARS-CoV-2

As neutralizing antibodies are an essential indicator of protective immunity, vaccines aim primarily to induce potent neutralizing antibodies specific to the viral S protein. RBD-specific IgG and neutralizing antibody responses were elicited by the Oxford-AstraZeneca (AZD1222/ChAdOx1) and Sputnik V (Gam-COVID-Vac-rAd26/rAd5) vaccines, measured >20 days after the first dose (75). These responses were boosted following a second vaccine dose (75). COVID-19 mRNA vaccine formulations have optimized the structure of the native S protein to allow prolonged exposure to immunogenic regions. The prefusion conformation of the native S protein has been targeted as a vaccine immunogen as it contains the epitopes for neutralizing antibodies (77). However, the native S protein tends to prematurely refold into its post-fusion state thereby hiding immunogenic regions (77, 78). In efforts to enhance the stability of the S protein prefusion conformation, vaccine developers have introduced a proline substitution to two consecutive residues (K986 and K987) in the S2 subunit between the central helix and first heptad repeat (77, 79). This engineered immunogen, termed the SARS-CoV-2 S-2P antigen, has been incorporated into mRNA COVID-19 vaccines and is expected to improve their immunogenicity (77, 79). Indeed, robust and persistent SARS-CoV-2 S protein-specific GC and Tfh cell responses were stimulated by COVID-19 mRNA vaccines, that strongly correlated with neutralizing antibody production (80, 81). Wisnewski and co-workers also demonstrated that COVID-19 mRNA vaccines elicited S antigen-specific IgA and IgG antibodies (82). The high neutralizing antibody titers elicited by these vaccines were detected in adults six months after receiving the second dose (83, 84). Interestingly, delaying the time (6-14 weeks) between first and second doses of the Pfizer-BioNTech (BNT162b2) mRNA and Oxford-AstraZeneca (AZD1222/ChAdOx1) vaccines resulted in higher neutralizing antibody levels compared to the three-week interval tested during vaccine licensing clinical trials (85–88). CD4^+^ and CD8^+^ T-lymphocyte responses were however slightly dampened with a longer time interval (87, 88). It has been postulated that these observations may be attributed to the longer time interval allowing more S-specific T-lymphocytes to differentiate into memory T-lymphocytes that respond more effectively upon re-exposure to the S protein (88). Delaying the time between vaccinations beyond three weeks also allows more people to receive their first dose, partially protecting them from severe COVID-19 and hospitalization; several countries including South Africa have opted for the longer interval regimen (89, 90). However, recent evidence suggests that vaccine-induced neutralizing antibody titers begin to decline six months after the second dose as plasmablasts induced by vaccines are short-lived and may not develop into long-lived plasma cells as is observed during natural infection (55, 71, 91).

Individuals with past SARS-CoV-2 infection, independent of disease severity, elicited a 10-to-45-fold higher neutralizing antibody titer than vaccinees without a history of infection after just a single dose of either the Pfizer-BioNTech (BNT162b2) or Moderna (mRNA-1273) mRNA COVID-19 vaccines (92, 93). Administration of a second dose did not alter this titer, suggesting that a single dose of an mRNA vaccine is sufficient to obtain peak antibody and memory B-lymphocyte levels in individuals with a history of COVID-19 infection (92, 94). Therefore, not only do COVID-19 vaccines mediate the production of neutralizing antibodies, but they may also maintain and boost titers in individuals with a history of infection. This is reassuring given that neutralization titers decay over time (60) and that protective immunity acquired from previous SARS-CoV-2 infection is not guaranteed given the varying immune responses elicited. However, a recent study demonstrated that individuals with immunity from previous infection alone have a 13-fold lower risk of breakthrough infection compared to those who received both doses of the Pfizer-BioNTech (BNT162b2) mRNA vaccine (95). Further investigation will be required to compare the humoral and cellular immune responses between these groups, as well as to the other COVID-19 vaccines, to effectively evaluate whether vaccination is advisable for people with a history of COVID-19 infection.

Studies investigating the immune response induced by COVID-19 vaccines have mainly focused on antibody-based responses, but T-lymphocyte memory cells and cellular immunity should also be further investigated as they are equally essential for the development of potent and reliable SARS-CoV-2 immunity (96). In addition, as a strategy to overcome SARS-CoV-2-induced innate immune suppression, it has been suggested that COVID-19 vaccines should be directed at innate immune memory or trained immunity (97). This could assist in reducing the risk of developing severe COVID-19 disease given that aberrant innate cytokine production by macrophages, DCs and neutrophils contributes to immunopathology and negatively influences subsequent adaptive immune responses.

## 2 SARS-CoV-2 Spike Protein

**Figure 2:**
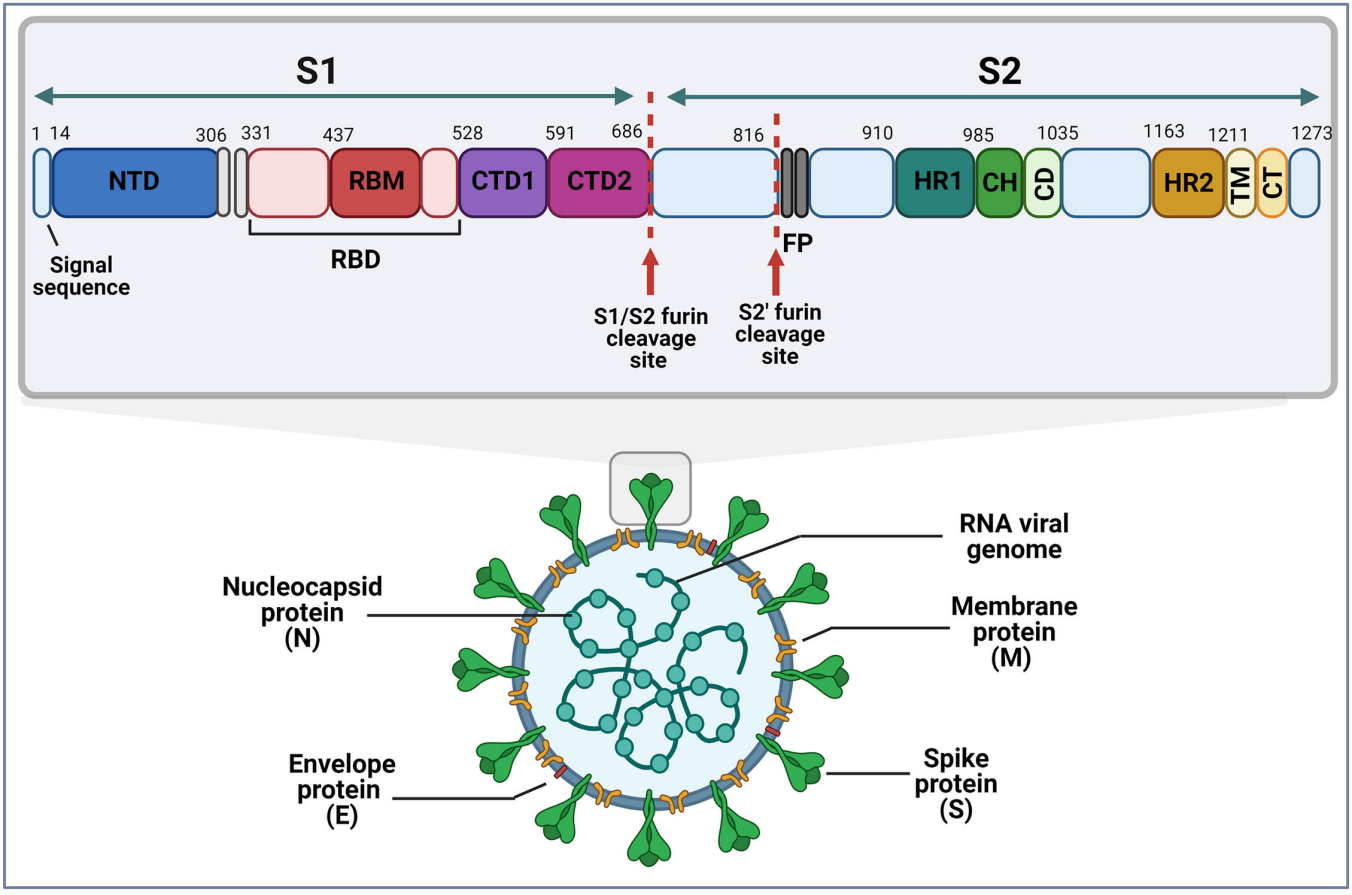
Schematic representation of the SARS-CoV-2 virion and the domain structure of its spike protein. Furin cleavage sites are as indicated, and numbers represent amino acid positions within the protein. (S1&S2, subunit 1 & 2; NTD, N-terminal domain; RBM, receptor binding motif; RBD, receptor binding domain; CTD1&CTD2, C-terminal domain; FP, furin peptide; HR1&HR2, heptad repeats; CH, central helix; CD, connector domain; TM, transmembrane domain; CT, cytoplasmic tail). Image created by PM using BioRender (https://biorender.com/).

SARS-CoV-2 contains four major viral structural proteins including the envelope (E), N, M and S proteins (**Figure 2**). The 1273 amino acid S protein consists of two subunits, an N-terminal S1 subunit of ~700 amino acids and a C-terminal S2 subunit of ~600 amino acids (**Figure 2**). The S1 subunit further consists of an N-terminal domain (NTD), an RBD and two C-terminal domains (CTD1 and CTD2). The RBD domain contains one or more species-specific receptor binding sub-domains or motifs (RBMs). The major domains of the S2 subunit are two heptad repeats (HR1 and HR2), a central helix (CH), fusion peptide (FP) and a connector domain (CD) which connects the spike to the virus membrane.

**Figure 3:**
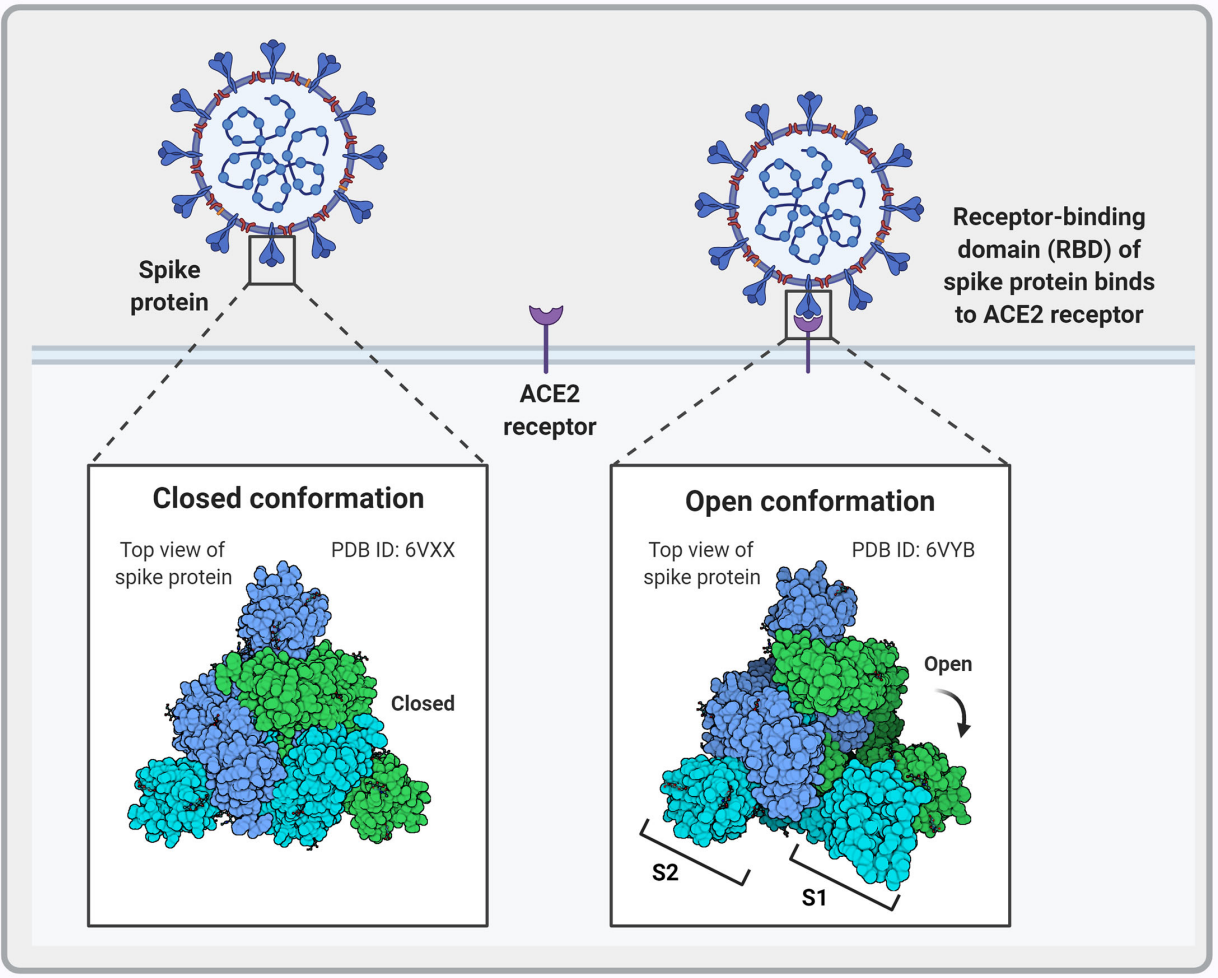
Schematic representation of the different conformational states of the SARS-CoV-2 spike protein. When in the closed conformation, the receptor binding domain (RBD) and its epitopes are hidden thereby contributing to immune evasion. In contrast, when subunit 1 (S1) dissociates from subunit 2 (S2) an open conformation is achieved that exposes the spike protein RBD and allows interaction with the host angiotensin-converting enzyme 2 (ACE2) to facilitate viral entry. Image created by PM using BioRender (https://biorender.com/).

The SARS-CoV-2 S protein interacts with the human angiotensin-converting enzyme 2 (ACE2) receptor to mediate viral attachment, fusion, and entry into host cells (**Figure 3**). Despite the low frequency of RBD in the open position, RBM ACE2 receptor affinity is high (98). Before S1 RBM host cell receptor binding, the S1 and S2 subunits are non-covalently bound. After host receptor binding, the S1 and S2 units dissociate under the influence of cell surface transmembrane serine protease 2 (TMPRSS2), endosomal protease cathepsin and other enzymes, priming the S2 subunit for virus host cell membrane fusion (99, 100). These enzymes present potential targets for intervention. Sequence analysis of SARS-CoV-2 S protein reveals the insertion of a furin cleavage site at the boundary between the S1 and S2 subunits (**Figure 2**). Cai et al. proposes that furin cleavage during virus packing may pre-activate the protein for membrane fusion independent of target cell receptor ACE2 binding or proteases and could lead to spontaneous S1 dissociation in mature virions (101).

The SARS-CoV-2 S protein is encoded by the 3.8 kb S gene on its positive-sense single-stranded RNA viral genome (102). Nucleotide changes in the SARS-CoV-2 genome that result in amino acid changes are predominantly in this region that codes for the S glycoprotein (57%), of which 38% are in the RBD (103). The focus of research thus far has therefore mainly been on amino acid alterations in the S protein since this protein mediates attachment to host cells and is the major target for neutralizing antibodies.

## 3 SARS-CoV-2 Variants

### 3.1 Emergence of SARS-CoV-2 Variants

**Figure 4:**
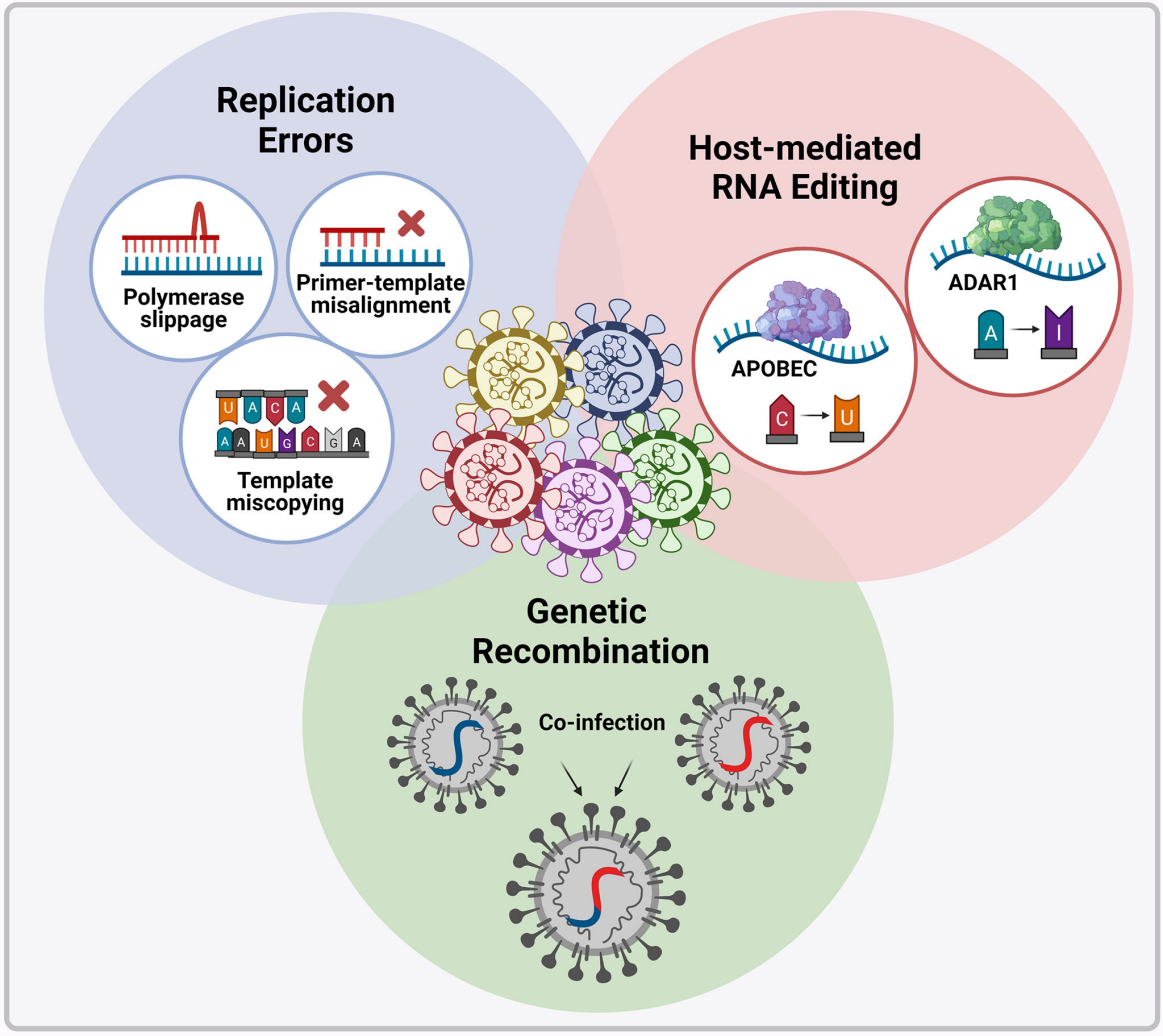
Molecular mechanisms introducing genomic alterations into the SARS-CoV-2 genome. Nucleotide changes can emerge naturally in the viral genome through the various replication errors shown. Apart from replication errors, host-derived RNA editing enzymes apolipoprotein B mRNA editing catalytic polypeptide-like enzyme (APOBEC) and adenosine deaminase RNA specific 1 enzyme (ADAR1) can introduce signature point substitutions [cytosine (C) to uracil (U) and adenosine (A) to inosine (I)] into the viral genome. Lastly, if two viral variants co-infect the same cell recombination can occur whereby the genetic material of the two variants are packages into a single virion. Image created by PM using BioRender (https://biorender.com/).

Variants (i.e. viral variations) occur through nucleotide changes that emerge naturally in the viral genome during replication (**Figure 4**), with these changes occurring at a higher rate in RNA viruses than in DNA viruses (104). However, the rate at which these nucleotide changes occur in CoVs is significantly lower than that of other RNA viruses since they possess an enzyme that corrects some errors made during replication (105). The SARS-CoV-2 non-structural protein 14 (nsp14) contains exoribonuclease (ExoN) activity that has been shown to have a “proofreading” effect; moreover, it has been demonstrated that inactivation of ExoN is detrimental to SARS-CoV-2 and MERS-CoV replication (106, 107). Advantageous genomic alterations with respect to viral replication, transmission, and immune evasion will increase in frequency in a population because of natural selection (108). Immune evasion occurs when genomic alterations make the immune response ineffectual due to its inability to recognize and eliminate the virus. On the contrary, genomic alterations that reduce viral fitness will be eliminated from the population of circulating viruses. Genomic alterations that have little effect on viral fitness can also increase or decrease in frequency by chance alone and contribute to the pool of circulating variants. Various treatment options (convalescent plasma, monoclonal antibodies (mAb), and vaccines) as well as environmental factors also act as selective pressures for new variants and contribute to the persistence of these variants. If variants demonstrably change the phenotype (virulence and transmission) of a virus, then they are referred to as a strain (104, 109).

Apart from replication errors, host-derived pressures such as RNA editing or RNA modification can also be essential for the formation of SARS-CoV-2 variants (**Figure 4**). Extensive cytosine-to-uracil and adenosine-to-inosine nucleotide substitution patterns have been observed upon SARS-CoV-2 genome analysis (110, 111). These patterns have been interpreted as signatures of RNA editing enzymes apolipoprotein B mRNA editing catalytic polypeptide-like enzyme (APOBEC) and adenosine deaminase RNA specific 1 enzyme (ADAR1) respectively (110, 111). Hosts have evolved these RNA editing enzymes as sensory viral innate immune mechanisms (112), however viruses can exploit these mechanisms for their evolutionary potential. The intra-host variability observed in COVID-19 patients has therefore been suggested to be driven by host RNA editing enzymes (113), but how SARS-CoV-2 manipulates these enzymes to introduce nucleotide changes into its viral genome remains unclear (112) (110, 111) (113).

SARS-CoV-2 plausibly originated as a result of cross-species recombination between bat and pangolin CoVs (114), and SARS-CoV-2 variants are likely to be emerging as a result of recombination (115, 116). Recombination occurs in cells infected with multiple variants, where the genetic material of two variants is packaged into a single virion (**Figure 4**). Recent evidence shows that two SARS-CoV-2 variants can infect one person simultaneously (117). These recombined virions may possess different pathogenic properties with serious implications for SARS-CoV-2 countermeasures, especially if the recombinants can escape both natural and vaccine-induced immunity (118). CoVs are known to have relatively high recombination rates (119); however, contradictory information has been reported on the recombination of SARS-CoV-2, and the overall extent and importance of ongoing recombination has not yet been resolved.

Accelerated viral evolution and prolonged shedding of replication-competent virus have been observed in immunocompromised individuals suffering from chronic SARS-CoV-2 infection (103, 120–122). Several studies have revealed strong selection pressure on SARS-CoV-2 during immune-based therapies such as convalescent plasma and mAb treatments, which have been shown to be associated with the emergence of viral variants with reduced susceptibility to neutralizing antibodies (103, 120, 123). However, the emergence of viral variants was also observed in immunocompromised human immunodeficiency virus (HIV)-infected patients without the administration of immune-based therapies (123, 124). Single nucleotide polymorphism (SNP) analysis identified several variations often resulting in amino acid changes in the S protein associated with immune evasion found in variants of concern (123, 124). The Beta variant, first discovered in South Africa, was hypothesized to have emerged through intra-host evolution in one or more individuals with prolonged viral replication, such as immunocompromised HIV patients. Intra-host evolution therefore seems to be more pronounced in immunocompromised populations which could serve as a long-term source of new SARS-CoV-2 variants, but it remains unclear whether underlying co-morbidities play a direct role in the formation of viral variants.

### 3.2 Currently Circulating SARS-CoV-2 Variants

SARS-CoV-2 variants are classified by the WHO into two types: variants of concern (VOC) and variants of interest (VOI). Several VOC have emerged from the original wild-type strain isolated in Wuhan since the outbreak first began in December 2019. According to the Centre for Disease Control (CDC), a VOC is one which has increased transmissibility, increased virulence, resistance to vaccine or acquired immunity from previous infection, and has the ability to elude diagnostic detection (125).

The D614G substitution was one of the earliest S protein modifications detected that quickly dominated in variants worldwide. Although variants with this amino acid substitution were more infectious (126–128), neutralization from convalescent serum was still effective (129, 130).

Variant Alpha (B.1.1.7) was first detected in late September 2020 and quickly became the predominant strain in the United Kingdom (UK) (131, 132). The Beta (B.1.351) variant, first detected in October 2020, became the dominant strain resulting in the second wave in South Africa (133). Similarly, variant Gamma (P.1) was detected in four Brazilians travelling to Japan in January 2021 (134) and was responsible for the resurgence in infections in Manaus, despite high levels of previous infection in the country (135, 136). Variant Delta (B.1.617.2), first detected in December 2020, was responsible for the massive rise in cases causing a second wave in India (137, 138) and breakthrough infections in multiple gatherings in the United States of America (139). Variant Omicron (B.1.1.529), recently designated VOC by the WHO, was first detected in November 2021 by world class genomic surveillance laboratories in South Africa and has been found in many countries around the world (140, 141). The emergence of these variants is concerning since they may affect viral transmissibility, virulence and rate of reinfection by escaping natural and vaccine-induced immunity (142).

Many other VOI have been reported which are only predicted to affect transmission, virulence and acquired or vaccine immunity. The VOI and variants being monitored include Epsilon (B.1.427/B.1.429) identified in California, Zeta (P.2) identified in Brazil, Eta (B.1.525) identified in Nigeria and the UK, Theta (P.3) identified in the Philippines, Iota (B.1.526/B.1.526.1) identified in New York, Kappa (B.1.617) and Delta Plus (B.1.617.2.1) identified in India, Lambda (C.37) identified in Peru and Mu (B.1.621) identified in Colombia (125, 143, 144). Further studies are required to elucidate their impact on the current COVID-19 climate.

**Figure 5:**
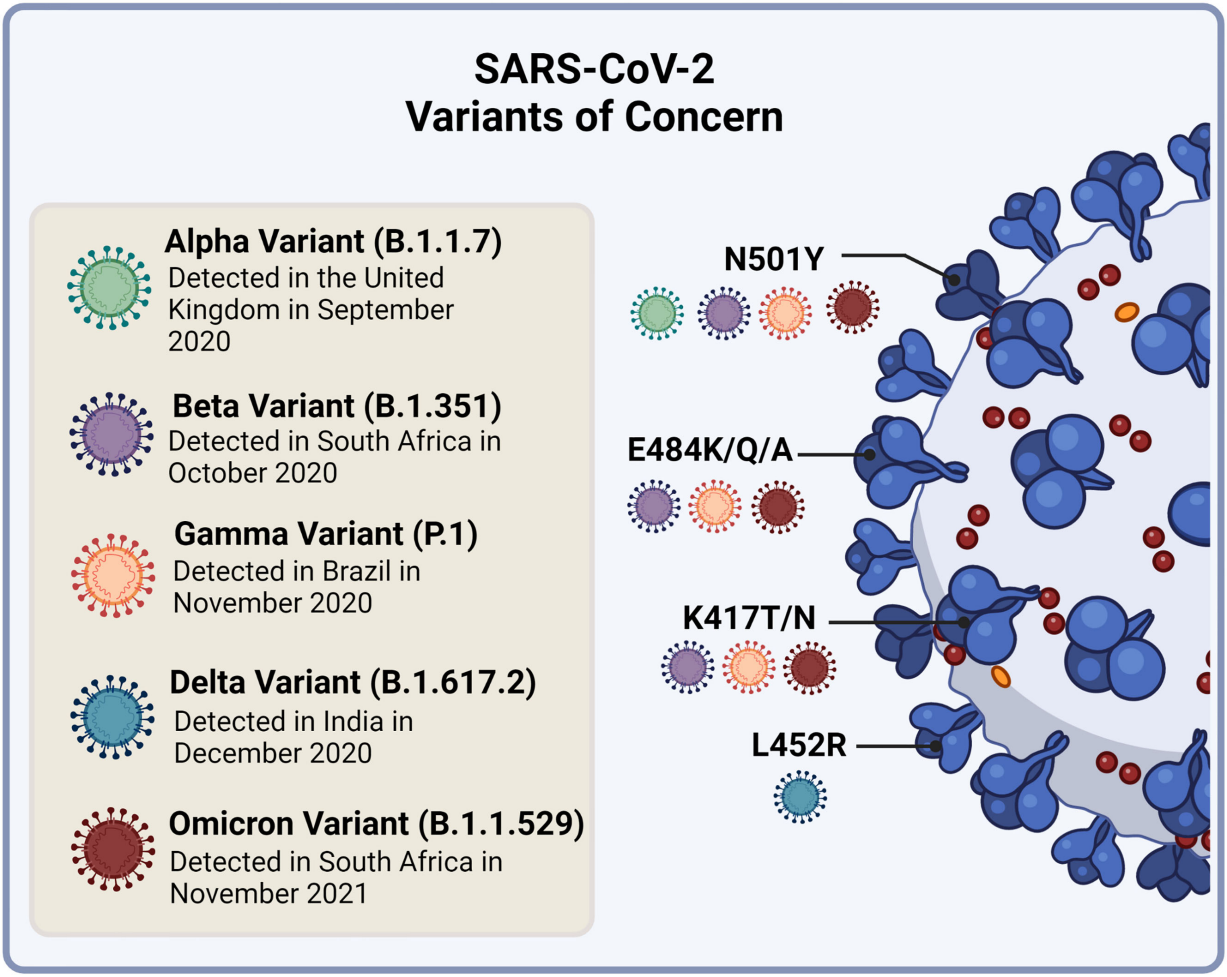
Currently circulating SARS-CoV-2 variants of concern and their receptor binding domain (RBD) amino acid substitutions of interest to virulence and immune evasion. The N501Y substitution is common to the Alpha, Beta, Gamma and Omicron variant strains. The E484K/Q/A and K417T/N substitutions are present in the Beta, Gamma and Omicron strains, while the L452R substitution is unique to the Delta variant. Image created by PM using BioRender (https://biorender.com/).

The SARS-CoV-2 VOC that have emerged share common signatures in their S protein, but each variant also displays novel changes. There are four main RBD amino acid substitutions which have been the focus of studies that assess virulence and immune evasion (**Figure 5**). The first is the N501Y found in the ACE2 binding site of the RBD and is common to Alpha, Beta, Gamma and Omicron variant strains (145, 146). The second and third substitutions, E484K/Q/A and K417T/N, are present in the Beta, Gamma and Omicron strains (145, 146). The fourth, L452R, is unique to the Delta variant (145). In addition, the Omicron variant has between 26-32 amino acid changes in the S protein which are the focus of investigations (147). This variant, although having a few substitutions in common with Beta and Delta, has a distinct evolutionary pathway (148).

### 3.3 Implications of Variants on Transmission and Virulence

As discussed earlier, the S protein attaches to the ACE2 receptor to facilitate entry of the virus into the host cell. Amino acid substitutions in the S protein, particularly in the RBD, could impact the virus’s ability to enter the host cell and present the greatest concern (**Table 1**).

**Table 1:** World Health Organization (WHO) SARS-CoV-2 variants of concern.

Variant	Spike Protein Alterations	Effect on Transmission	Effect on Virulence
Alpha	69/70 Δ	Increased	Increased
20I/501Y.V1/B.1.1.7	144Y Δ		
	N501Y		
	A570D		
	D614G		
	P681H		
	T716I		
	S982A		
	D1118H		
Beta	L18F	Increased	Increased
20H/501Y.V2/B.1.351	D80A		
	D215G		
	Δ242-244del		
	R246I		
	K417N		
	E484K		
	N501Y		
	D614G		
	A701V		
Gamma	K417T	Increased	Increased
P.1/20J/501Y.V3/B.1.1.248	E484K		
	N501Y		
	D614G		
	H655Y		
	L18F		
	T20N		
	P26S		
	D138Y		
	R190S		
	T1027I V1176F		
Delta	L452R	Increased	Increased
21A/B1.617.2	D614G		
	P681R		
	T19R		
	T478K		
	D950N		
	R158G		
	156,157 del		
Omicron 21K/ B.1.1.529	A67V	Increased	Unknown
	Δ69-70		
	T95I G142D/Δ143-145 Δ211/L212I ins214EPE G339D		
	S371L		
	S373P		
	S375F		
	K417N		
	N440K		
	G446S		
	S477N		
	T478K		
	E484A		
	Q493R		
	G496S		
	Q498R		
	N501Y		
	Y505H		
	T547K		
	D614G		
	H655Y		
	N679K		
	P681H		
	N764K		
	D796Y		
	N856K		
	Q954H		
	N969K		
	L981F		

N501Y is common amongst the Alpha, Beta and Gamma variants and has been shown to increase cell infectivity in animal models (149). The asparagine at position 501 (N501) is one of six essential amino acids involved in ACE2 receptor binding (150) and its substitution with tyrosine (Y) has been shown to increase binding affinity to the host receptor (151). This has likely contributed to the increased transmissibility and higher viral burden seen in both the Alpha (132, 152) and Beta (133) variants. Initial analysis based on matched case control studies in the UK indicated that the Alpha variant was not significantly associated with an increased risk of hospitalization or mortality in infected individuals. However, further analysis indicates that the variant is associated with an increase in severity and consequently a rise in mortality rate of up to 61% (152–154). The Alpha variant also contains the P681H substitution which is found in an area adjacent to the furin cleavage site that is known to be important in infection and transmission (155, 156). The ΔH69/V70 deletion also present in the Alpha variant enhances viral infectivity *in vitro* and has been linked to certain commercial kits failing to detect the S glycoprotein (157).

Amino acid substitutions in the Beta variant S protein are more extensive than in Alpha presenting 10 changes with three in the RBD. The RBD-ACE2 interaction complex has been analyzed structurally using *in silico* methods to assess the impact of the N501Y, K417T and E484K substitutions (158). The N501 residue is important for ACE2 interaction while K417 and E484 are not predicted to play a major role. The latter two residues may in fact reduce binding affinity, which shows that the increase in transmissibility seen in the Beta variant is due to N501Y or other alterations in the virus. In October 2020, the Beta variant accounted for 11% of SARS-CoV-2 virus sequenced in South Africa but by December of that year 87% of cases were due to this variant (159). In Cape Town a threshold of 100 000 cases was reached 50% more rapidly in the second wave with the Beta variant than in the first wave caused by the wild-type variant. Preliminary analysis also indicated that there was a higher mortality rate in the second wave although this may be due to the overwhelming impact on the health care system during that time. Pearson et al. (160) confirmed these results using a calibrated model which showed that this variant has increased transmissibility and virulence.

The Gamma variant was responsible for the surge in infections in Brazil in late 2020 that pushed the healthcare system to the verge of collapse. This occurred despite a previously high level of infection during the first wave indicating that the variant was not susceptible to naturally acquired immunity. Gamma contains 17 amino acid substitutions including a similar trio in the S protein found in Beta. Computational modelling demonstrated that the variant was 1.4-2.2 times more transmissible than the wild-type variant (135, 161). Reports have indicated that the Gamma variant may be more severe with a greater number of younger individuals presenting with advanced disease and succumbing to the virus (162, 163). Preliminary studies comparing the case fatality rates in the first and second waves showed that the death rate in individuals aged 20-39 was 2.7 times higher in the second wave and 1.15 times higher in the general population overall (164).

A dominant variant with eight S protein changes, the Delta variant, was responsible for the severe second wave that affected India earlier this year and has now also spread widely to other countries (138). This variant resulted in a devastating third wave in South Africa and rapidly displaced the Beta variant within three months of introduction into the country: this was attributed to the 46% transmission advantage compared to Beta (165). This variant also displaced the Alpha strain in the UK forcing government to delay the reopening of society planned for in June 2021 due to an increased number of infections particularly in younger, unvaccinated population groups (166). An amino acid substitution found in the RBD, L452R, and one in the S protein furin cleavage site, P681R, have been implicated in the increased transmission associated with this variant. The P681R substitution, also found in the Alpha variant, was demonstrated to increase fusion activity to the host receptor which could possibly increase infectivity and transmission rates (137). However, the P681R mutation has previously been identified in variants that did not increase in frequency as efficiently, indicating that a cumulative number of mutations may be responsible for this increase in transmission. Preliminary epidemiologic and genomic data have shown that the Delta variant is more transmissible than the original strain as well as other variants (167, 168). The risk of hospitalization was also found to be double that of the Alpha variant (169), with a recent study indicating that hospitalized patients infected with Delta had more severe disease and a higher in-hospital mortality rate (170).

Many groups are analyzing epidemiological data to establish the effect of the Omicron variant on transmission and virulence. Preliminary reports suggest that the variant may pose an increased risk of re-infection with case numbers increasing steeply in South Africa (171). The Omicron variant comprised 76% of samples sequenced in November 2021 in South Africa where the Delta variant had previously dominated tested sequences (172). Although some early indications from South African hospitals suggest that the Omicron variant causes a milder disease form in comparison to Delta, it is still too early to establish its effect on virulence (173).

### 3.4 Implications of Variants on Immunity and Vaccines

The current vaccines are designed on the premise that they elicit neutralizing antibodies against the S protein from the SARS-CoV-2 wild-type strain. The presence of S protein antibodies is strongly associated with protection against COVID-19 (60, 174). The emergence of viral variants may reduce the efficacy of vaccines since neutralizing antibodies elicited against the wild-type S protein strain may not recognize S protein variants. Furthermore, variants capable of re-infection, bypassing immunity acquired from previous infection, can precipitate a rise in cases that can overwhelm the healthcare system, as was observed in Brazil at the end of 2020. The efficacy of therapeutic and prophylactic mAb therapies, pre-authorized for emergency use, can also be affected by these variants.

Early reports indicated that the Alpha N501Y substitution does not compromise serum neutralization potential after vaccination (175, 176). Muik and co-workers assessed sera from 16 participants who had received the mRNA-based Pfizer-BioNTech vaccine (BNT162b2) against the original strain and pseudovirus S only portion of Alpha (177). The study showed that sera had similar neutralization potential against both the Alpha S protein and the wild-type virus.

The second alteration of concern located in the S protein, the 69-70 deletion, is associated with reduced neutralization by SARS-CoV-2 human convalescent serum (157) and viral escape in immune-compromised individuals (132). However, sera from both convalescent individuals and vaccine sera retain the ability to cross neutralize Alpha, albeit with a slightly reduced effect (72, 178–180). *Post-hoc* analysis from the safety and efficacy study of the protein-based Novavax (NVX-CoV2373) trial indicated that the vaccine was still highly effective (86.3%) against the Alpha variant (181). This suggests that the immune response from previous infection with the wild-type virus or from vaccination will still provide protection against the Alpha variant. Wang and co-workers, have shown however that the Alpha NTD may be resistant to mAbs currently used for therapy (72).

The E484K substitution has been identified in numerous *in vitro* laboratory studies to escape neutralization from both convalescent serum and mAbs (182, 183). Unsurprisingly, both the Beta and Gamma variants resist neutralization by convalescent serum from the original strain as well as antibodies from COVID-19 vaccinated individuals (72, 129, 184).

Neutralization assays using sera from recipients of the AstraZeneca (AZD1222/ChAdOx1) vaccine demonstrated a ≤86-fold reduction in neutralization activity with activity levels being undetectable against the Beta variant, resulting in immune escape (185). However, serum from recipients of the Moderna (mRNA-1273), Pfizer-BioNTech (BNT162b2) and Sinopharm (BBIBP-CorV) vaccines displayed a much lower ≤6.5, ≤8.6 and ≤1.6-fold reduction respectively in neutralization against the Beta variant (159). Covaxin (BBV152), an inactivated virus-based vaccine, also demonstrated a decreased neutralizing antibody titre in comparison to the D614G strain (186). Preliminary data from Novavax (NVX-CoV2373) trails showed that the vaccine can still effectively protect against the Beta variant (60%) albeit at a lower level than that of Alpha and the D614G strain (187).

A virus neutralization assay performed by Ikegame and co-workers evaluated the neutralization activity of sera from individuals vaccinated with the Sputnik V (Gam-COVID-Vac-rAd26/rAd5) vaccine against a replication-competent version of the Beta variant (178). Virus containing only the E484K substitution showed a moderate decrease in serum neutralization (2.8-fold) as compared to virus containing all the Beta substitutions, which displayed a more significantly reduced level (8.6-fold) of neutralization (178). Although the E484K substitution provides some resistance to neutralization, it is the cumulative changes in the Beta variant that contributes to the high level of resistance seen in response to vaccine induced immunity. This was further illustrated by Wibmer and co-workers who demonstrated the role of K417N and NTD changes in the Beta variant resistance to antibodies (184).

Since Gamma and Beta share common alterations in the RBD, concerns regarding the Gamma variant’s resistance to mAbs, vaccine or acquired immunity are not unfounded. Despite a previously high level of infection during the first wave in Brazil, the Gamma variant displayed an increase in cases of reinfection in the second wave which indicated that the variant was not susceptible to acquired immunity (135, 136). Early reports using computational modelling indicated that the Gamma variant could evade 25-61% of immunity acquired from previous infection (135).

Efficacy and viral neutralization trials for the Gamma variant showed a 6.7- and 4.5-fold decrease in activity with the Pfizer-BioNTech (BNT162b2) and Moderna (mRNA-1273) vaccines respectively (159). However, despite having changes that are similar to the Beta variant, the Gamma variant is less resistant to neutralization (188). Analysis of the Gamma variant against mAbs used in clinical therapy showed a pattern of significant escape comparable to the Beta variant. But neutralization with convalescent serum only had a 3.1-fold reduction in comparison to a 13-fold reduction with the Beta variant. The Gamma variant was also less affected by vaccine serum. The reduction in neutralization capacity of the Gamma variant was not as severe as the Beta variant (189) and has similar neutralization capacity to that of the Alpha variant (188). However, Fernández and co-workers did find that plasma from individuals who received two doses of the Sinovac (CoronaVac) vaccine had a decreased neutralization efficacy of 31.8% for Alpha and 59.1% for Gamma *in vitro (* 190).

The L452R substitution in the Delta variant is found in an area of the RBD that is known to be resistant to mAbs (191). Studies have indicated that the Delta variant is resistant to certain neutralizing antibodies including the currently used therapeutic mAb, Bamlanivimab (192). The study also demonstrated the effect of serum from convalescent patients which was 4-fold less potent against Delta than Alpha (192). Liu and co-workers also showed decreased neutralization potential of convalescent serum from individuals infected previously with the Beta variant, which indicated the potential for reinfection in South Africa (193). Planas and co-workers also assessed the effect of serum from individuals vaccinated with the Pfizer-BioNTech (BNT162b2) and AstraZeneca (AZD1222/ChAdOx1) vaccines on the Delta variant. Serum from those vaccinated with one dose barely inhibited the virus (192). After two doses of the respective vaccines, neutralization response was 3-5-fold lower than against Alpha (192). However, there is only a minimal decrease in effectiveness against symptomatic disease after a single dose and even less of a difference after two doses of the Pfizer-BioNTech (BNT162b2) and AstraZeneca (AZD1222/ChAdOx1) vaccines, which is comparable to the Alpha variant (194). The Janssen Pharmaceuticals (Ad26.COV2.S) non-replicating viral vector vaccine also showed a serum neutralization potential of 1.6-fold lower than the D614G lineage, although, the decrease in serum neutralization potential for Delta was still less than the decrease observed for the Beta (3.6-fold) and Gamma (3.4-fold) variants (195). Few studies have assessed the neutralization potential of the Delta variant against inactivated and protein-based vaccines. However, Yadav and co-workers did indicate that the serum from vaccine participants who received Covaxin (BBV152) and serum from convalescent individuals showed a significant reduction in neutralization potential for both Beta and Delta variants, with the latter being less so (186). This L452R substitution has also been shown *in vitro* to escape HLA-A24-restricted cytotoxic T-lymphocyte mediated cellular immunity (196) which is concerning since COVID-19 severity has been linked to a weaker virus-specific cellular immune response (197).

The Delta variant has continued to evolve and acquire new variants of concern such as the K417N substitution first identified in the Beta variant. This resulted in the emergence of the Delta Plus (B.1.617.2.1) strain, which is being closely monitored (144).

The Omicron VOC has twice the number of mutations found in Delta (198) which has heightened fears that this variant may reduce the efficacy of current vaccines and monoclonal antibody treatments. Pulliam and co-workers performed a retrospective analysis on epidemiological data which indicated that the Omicron variant is associated with an increased ability to evade immunity from previous SARS-CoV-2 infection (199). Computational predictions have further indicated that the structural changes may decrease antibody interaction but not completely evade neutralizing antibodies (200).

Preliminary data released by Pfizer-BioNTech demonstrates that a third dose of the Pfizer-BioNTech (BNT162b2) vaccine neutralizes the Omicron variant whereas only two doses have a significantly decreased neutralization titer (201). Three doses increased the neutralization titer by 25-fold when compared to just two doses (201). Similarly, preliminary data from South Africa also showed that the neutralization capacity of plasma from Pfizer-BioNTech (BNT162b2) vaccinated individuals showed a 41-fold decrease against the Omicron variant but previous infection together with vaccination increases the neutralization level (202). Vaccinated individuals may, however, still be protected against severe disease with two doses as the CD8+ T-lymphocyte epitopes on the S protein are not significantly affected by the amino acid substitutions present in the Omicron variant (201). Furthermore, although Omicron illustrated a more severe immune escape than the Beta variant in vitro, the ability to completely evade immunity from previous SARS-CoV-2 infection and vaccination was not observed (202).

## 4 Future Concerns and Considerations

### 4.1 Future Treatments and Vaccine Regimens

Presently, circulating SARS-CoV-2 VOC are posing challenges for mAb therapies and vaccine manufacturers due to possible resistance and vaccine ineffectiveness, respectively. There are concerns about whether these approaches will confer protection against new variants.

Recognizing that mAbs are not a long-term solution, they could nonetheless potentially benefit vulnerable populations that are at a higher risk of contracting moderate/severe disease and respond poorly to vaccination. mAb therapies could be used as an early intervention to counter the devastating impact of the virus on vulnerable populations. A recent study identified a new mAb (named S2X259) with broadly neutralizing effects that targets a highly conserved region of the RBD called antigenic site II (203). This region is usually inaccessible because of the RBD conformation and therefore a low fraction of antibodies generally targets this site in infected individuals. S2X259 reacted with 29 of 30 S proteins of sarbecoviruses, including SARS-CoV-2 and its new variants. The study also showed cross-reactivity of S2X259 with bat sarbecoviruses, further demonstrating its wide applicability as a broadly neutralizing antibody (203). Binding of this antibody was not hindered by alterations in the RBD, which are present in the Alpha, Beta and Gamma variants. The epitope that the antibody binds to is conserved in all circulating SARS-CoV-2 variants. In addition, it does not target the 417 or 484 residues in new variants, and therefore might explain its potency against different variants (203). The challenges with mAbs include the complexity of their production, which would result in a limited supply initially, and temporary immune protection offered by antibodies in general, since long-term protection has not been demonstrated.

Vaccines offer protection without the risk of infection and subsequent severe symptoms, and remain the best strategy to reduce disease burden and safely acquire immune protection against SARS-CoV-2. Updating or developing new COVID-19 vaccines may therefore be necessary to control viral variants. Research led by Moore and Sigal showed that infection with the Beta variant triggered antibodies that fended off old and new variants (204). According to Moore, it is possible that these antibodies recognize features of the viral S protein that are similar between variants. New COVID-19 vaccines could be made more resilient than existing vaccines by targeting multiple S protein epitopes that are not prone to genomic alteration. This will require that genetic changes in SARS-CoV-2 that may influence vaccine effectiveness be closely monitored. Apart from the S protein, future COVID-19 vaccines should also include other highly immunogenic viral proteins, such as the N protein (38), thereby offering broader protection.

Current mRNA, protein and viral vaccines could be updated for VOC by replacing older variants of the S protein with those of emerging variants. It might be possible to include both old and new forms of the S protein in a single vaccine, termed a multivalent vaccine (205). It is however still unknown how people who have already received current vaccines would react to new vaccines. It has long been observed that people tend to elicit stronger immune reactions to the first variant of a pathogen. This could mean that modified/updated vaccines could trigger a muted immune response in which a second vaccination against a new variant may not trigger a response against the new variant, but would rather result in a booster effect of the old response (205). The question remains as to how often these vaccines would need to be updated to remain effective and keep the virus under control.

Breakthrough infections among fully vaccinated individuals have recently been reported (206, 207). Waning immunity approximately six months post-vaccination rather than reduced effectiveness of vaccines against VOC is most likely the cause (208). Although vaccinated individuals are at risk of reinfection, transmission rates are lower among vaccinated compared to unvaccinated individuals (209). Vaccinated individuals further show decreased disease severity and are more likely to recover and less likely to require hospitalization (208, 210). Booster shots are presently being administered to fully vaccinated people in some countries and have been shown to reduce severe COVID-19 and SARS-CoV-2 infections (211). A recent study indicates that current vaccines provide sufficient protection against severe disease (212). Scientists and the WHO are therefore reluctant to suggest booster shots for all, especially since globally, many people are yet to receive their first dose. However, if booster shots are approved, the timing of administration should be evaluated to ensure maximum protection as this has been shown to influence the kinetics and magnitude of the immune response elicited.

Several countries have started combining COVID-19 vaccines, referred to as a heterologous vaccine strategy (213, 214). Individuals who had previously received the Oxford-AstraZeneca (AZD1222/ChAdOx1) vaccine were given the Pfizer-BioNTech (BNT162b2) mRNA vaccine after eight weeks (215). Vaccinees elicited more robust immune responses that generated more antibodies as well as memory B cells than the control group who received the Oxford-AstraZeneca vaccine alone (213–215). The United Arab Emirates recently started administrating Pfizer-BioNTech (BNT162b2) booster shots to individuals six months after the second Sinopharm (BBIBP-CorV) shot (216). It remains however to be established how effective this vaccination strategy is against the VOC.

Rare vaccine side effects may occur as a result of the immune system reacting to the viral-vector present in some vaccines, as is seen with repeated doses of the Oxford-AstraZeneca (AZD1222/ChAdOx1) vaccine. Enhanced side effects were suspected to occur when mRNA vaccine booster shots were first introduced. However, early data suggests that side effects are similar to the ones experienced after the second dose of mRNA vaccines (217). Adverse reactions may be influenced by genetic as well as other host risk factors and further investigation is required to determine how host-vaccine interactions may affect the success of re-vaccination strategies.

### 4.2 Correlate of Protection

Evaluating the efficacy of existing vaccines against VOC is paramount for controlling viral variants as well as ensuring effective immunization, and should be extended to include VOI. Current clinical trials evaluating vaccine efficacies against variants are however time consuming and costly. As has been seen with vaccine rollout in South Africa, vaccine efficacy results are promptly needed to make vaccine decisions and it is therefore impractical to continue repeating clinical trials for each newly emerged variant (218). This stresses the urgent need for a correlate of protection for COVID-19 vaccines that will allow vaccine efficacy results against pre-existing variants to be translated to newly emerged variants (218, 219). A correlate of protection is defined as an immune biomarker that statistically represents the level of protection a person has acquired against a virus (220). A correlate of protection will assist in overcoming challenges associated with Phase III vaccine licensure of new vaccine candidates or modified vaccines as well as aid healthcare providers with management of immunocompromised individuals (219). Furthermore, a protective threshold can be established that may assist in clarifying whether natural SARS-CoV-2 infection confers sufficient protective immunity or if vaccine boosters are required. Officials will therefore be able to effectively monitor the progress of vaccine rollout, provide a more accurate estimation of the level of herd immunity that has been achieved and identify a potential need for interventions.

The WHO has had extensive discussions on the clinical and statistical considerations involved in defining a correlate of protection (221). Similarly, Karim (218) identified four essential requirements for stratifying a correlate of protection based on disease severity. Studies have also aimed to identify candidate correlates of protection in addition to neutralizing antibodies (222–224). There is currently no standardised correlate of protection, however, by developing a deeper understanding of immune responses coupled with a global repository of serum samples, data sharing (225) and a dedicated research agenda, it is hoped that a reliable immune biomarker will be established.

### 4.3 Possible Receptor Switching

RNA viruses, such as HIV, can utilize more than one host receptor to mediate cellular invasion. Similar to other CoVs such as MERS-CoV and SARS-CoV (226, 227), it is suspected that SARS-CoV-2 may also develop the ability to infect host cells *via* the S protein binding to receptors other than its primary receptor of entry as the virus continues to evolve fitness-enhancing variations. Genome analysis of SARS-CoV-2 indicated that ACE2 is the receptor used for viral entry (228) and this was confirmed with functional studies using transgenic mice (229). However, *in vitro* studies have suggested that the transmembrane glycoprotein CD147 could serve as an alternative receptor for SARS-CoV-2 (230). This glycoprotein functions as a receptor for cyclophilin A which plays a role in the inflammatory response by acting as a chemotactic factor for leukocytes and additionally activates antiviral responses (231). Although evidence is still required for the role of integrins, which are CD147 interacting proteins, they have also been hypothesized to be SARS-CoV-2 host entry receptors (232). Neurophilin (NRP) 1 and NRP2 contain a domain sequence which according to molecular modelling and *in vitro* studies, could serve as a binding site for the SARS-CoV-2 furin cleavage site thereby acting as a possible co-receptor for virus entry (233, 234).

*In vitro* studies on a murine CoV illustrated that a few additional amino acid changes in the S protein could lead to a change in receptor usage (235). Recent studies have indicated the use of alternative receptors by SARS-CoV-2 with an E484D S protein substitution *in vitro (* 236). H522 lung adenocarcinoma cells, which do not express ACE2, were infected by this variant through an unknown receptor with the aid of heparan sulphate proteoglycans. The Alpha and Beta VOC as well as Epsilon VOI still maintained robust cell entry *via* ACE2 and fusion *via* TMPRSS2 when studied in cell lines (237). However, additional work is required to assess the variants’ affinity for alternative receptors.

## 5 Conclusion

The COVID-19 pandemic has devastated countries both economically and socially, with a constantly evolving population of SARS-CoV-2 viruses that will likely remain a part of our lives for many years to come. In the last two decades, three CoV sub-species have crossed over to humans and the latest pandemic is unlikely to be the last. The scientific community must continue to improve surveillance and monitoring strategies to prevent these occurrences from having such damaging effects in the future.

Global routine surveillance of SARS-CoV-2 variants and their effects on virulence and currently used therapeutics will allow scientists to assess if vaccines and other therapies are required to be updated periodically. New variants may penetrate herd immunity and infect unvaccinated individuals or facilitate vaccine escape, which can predispose these individuals to severe disease or death. However, most studies have suggested that vaccines are still effective against the currently circulating variants and can protect against severe to moderate disease outcomes. Evidence supporting the use of even single vaccination doses in preventing severe disease from the Delta variant in the UK has highlighted the need for faster vaccination provision and rollout in poorer regions such as Africa. It is hoped that the in-house manufacturing of COVID-19 vaccines will assist South Africa as well as the rest of the African continent to achieve this goal. In addition, more studies are needed to evaluate the reason behind breakthrough infections and the possibility of waning immunity to SARS-CoV-2 as well as the role that booster vaccine doses could play in prevention.

Future studies should assess the cell mediated immune responses to SARS-CoV-2 variants since both endogenous and exogenous T-cell mediated pathways may provide a broad-spectrum protection that is precluded by humoral immunity. To reduce the risk of new and potentially more deleterious variants from emerging, health authorities should focus on vaccinating individuals as rapidly as possible and should continue to emphasize the importance of social distancing and mask wearing. A multipronged treatment approach should continue to be implemented in countries such as South Africa that has a high prevalence of co-morbidities such as HIV which may contribute to the emergence of variants. This will not only save lives, but also provide limited room for the virus to evolve. While it may not be possible to predict what the next VOC will be, we can learn from past experiences and challenges to better cope with the situation at hand.

## Author Contributions

All authors contributed equally to the concept and preparation of the manuscript. PM and MP completed the final preparation and editing of the manuscript. PM created the figures. All authors contributed to the article and approved the submitted version.

## Funding

This work has been supported by the South African Medical Research Council (SAMRC) Extramural Unit for Stem Cell Research and Therapy University of Pretoria through the Institute for Cellular and Molecular Medicine. PM receives funding from the National Research Foundation (NRF) Postgraduate Scholarship (MND200610530106). FB receives funding from the SAMRC under the Internship Scholarship Programme from funding received from the South African National Treasury. KP receives funding from the SAMRC (A1A982) and the DAAD-NRF Doctoral Scholarship (123306). AS and JM receive funding from the Bill & Melinda Gates Foundation (INV-022216).

## Author Disclaimer

The content hereof is the sole responsibility of the authors and do not necessarily represent the official views of the SAMRC or the funders.

## Conflict of Interest

The authors declare that the research was conducted in the absence of any commercial or financial relationships that could be construed as a potential conflict of interest.

## Publisher’s Note

All claims expressed in this article are solely those of the authors and do not necessarily represent those of their affiliated organizations, or those of the publisher, the editors and the reviewers. Any product that may be evaluated in this article, or claim that may be made by its manufacturer, is not guaranteed or endorsed by the publisher.
